# Glomerular Endothelial Cells Are the Coordinator in the Development of Diabetic Nephropathy

**DOI:** 10.3389/fmed.2021.655639

**Published:** 2021-06-18

**Authors:** Tingting Li, Kaiyuan Shen, Jiawei Li, Susan W. S. Leung, Tongyu Zhu, Yi Shi

**Affiliations:** ^1^Key Laboratory of Organ Transplantation, Zhongshan Hospital, Fudan University, Shanghai, China; ^2^Institute of Clinical Science, Zhongshan Hospital, Fudan University, Shanghai, China; ^3^Department of Neurology, Zhongshan Hospital, Fudan University, Shanghai, China; ^4^Department of Urology, Zhongshan Hospital, Fudan University, Shanghai, China; ^5^Department of Pharmacology and Pharmacy, Li Ka Shing Faculty of Medicine, The University of Hong Kong, Hong Kong, China

**Keywords:** angiogenesis, glomerular endothelial dysfunction, single cell RNA analysis, diabetic nephropathy, cell crosstalk

## Abstract

The prevalence of diabetes is consistently rising worldwide. Diabetic nephropathy is a leading cause of chronic renal failure. The present study aimed to explore the crosstalk among the different cell types inside diabetic glomeruli, including glomerular endothelial cells, mesangial cells, podocytes, and immune cells, by analyzing an online single-cell RNA profile (GSE131882) of patients with diabetic nephropathy. Differentially expressed genes in the glomeruli were processed by gene enrichment and protein-protein interactions analysis. Glomerular endothelial cells, as well as podocytes, play a critical role in diabetic nephropathy. A subgroup of glomerular endothelial cells possesses characteristic angiogenesis genes, indicating that angiogenesis takes place in the progress of diabetic nephropathy. Immune cells such as macrophages, T lymphocytes, B lymphocytes, and plasma cells also contribute to the disease progression. By using iTALK, the present study reports complicated cellular crosstalk inside glomeruli. Dysfunction of glomerular endothelial cells and immature angiogenesis result from the activation of both paracrine and autocrine signals. The present study reinforces the importance of glomerular endothelial cells in the development of diabetic nephropathy. The exploration of the signaling pathways involved in aberrant angiogenesis reported in the present study shed light on potential therapeutic target(s) for diabetic nephropathy.

## Introduction

The prevalence of diabetes keeps rising worldwide (1). Diabetes and diabetes-induced complications remarkably affect life quality and reduce life span compared with the non-diabetes population, although many advances have been made in the early diagnosis and clinical treatments (1, 2). Diabetes-induced complications include retinopathy, nephropathy, and neuropathy. Among them, diabetic nephropathy is a leading cause of chronic renal failure. Patients with diabetic nephropathy present albuminuria (<300 mg per day) at an early stage and later develop proteinuria, leading to renal failure (3, 4). Pathological changes in diabetic nephropathy include glomerular capillary widening, glomerular basement membrane thickening, mesangial matrix expansion, arteriolar hyalinosis, and glomerulosclerosis.

Glomeruli are a tight cluster of capillaries consisted of endothelial cells, podocytes, and mesangial cells. Inside the glomerulus, endothelial cells, podocytes, and glomerular basement membrane are fundamental structures for glomerular filtration. Mesangial cells are supporting cells functioning as pericytes and vascular smooth muscle cells. In diabetic patients, podocyte foot process changes are consistently observed. Since preservation of these changes reduces urinary protein excretion and improves kidney function (5), podocyte injury is considered to be a vital feature of diabetic nephropathy. On the other hand, the role of glomerular endothelial cells has been intensively studied in the last decade (6, 7). Diabetes-induced glomerular endothelial dysfunction presents the destruction of fenestrated endothelial integrity, increased cell proliferation, and immature angiogenesis, as well as an increased endothelial-to-mesenchymal transition (8). Of note, immune cells, including macrophages, T lymphocytes, B lymphocytes, plasma cells, and dendritic cells, are all involved in the development of diabetic nephropathy (9–16). Thus, it is critical to consider the importance of cellular crosstalk inside glomeruli in the progress of diabetic nephropathy since glomeruli are a fine-tuning functional unit.

Single-cell sequencing, the updated version of the next-generation sequencing technologies, provides a high resolution of cell differences in microenvironments. The use of single-cell sequencing have led to the identification of novel cells and a better understanding of specific cells in comprehensive microenvironments in developmental biology (17, 18), neurology (19), oncology (20), immunology (21, 22), cardiovascular research (23, 24), infectious disease (23, 25) as well as microbiomes (26). The online single-cell sequencing data (GSE131882) have identified fifteen types of cells, including parenchymal cells and immune cells, in the renal cortex of diabetes patients (27). The present study focused on crosstalk inside human diabetic glomeruli by subsetting the genomic data of glomerular endothelial cells, podocytes, mesangial cells, and immune cells. It was designed to investigate the role of glomerular endothelial cells under diabetic conditions, with special attention being paid to the interactions of endothelial cells with other cells inside glomeruli in the progress of diabetic nephropathy.

## Methods

### Data Sources

**Figure 1:**
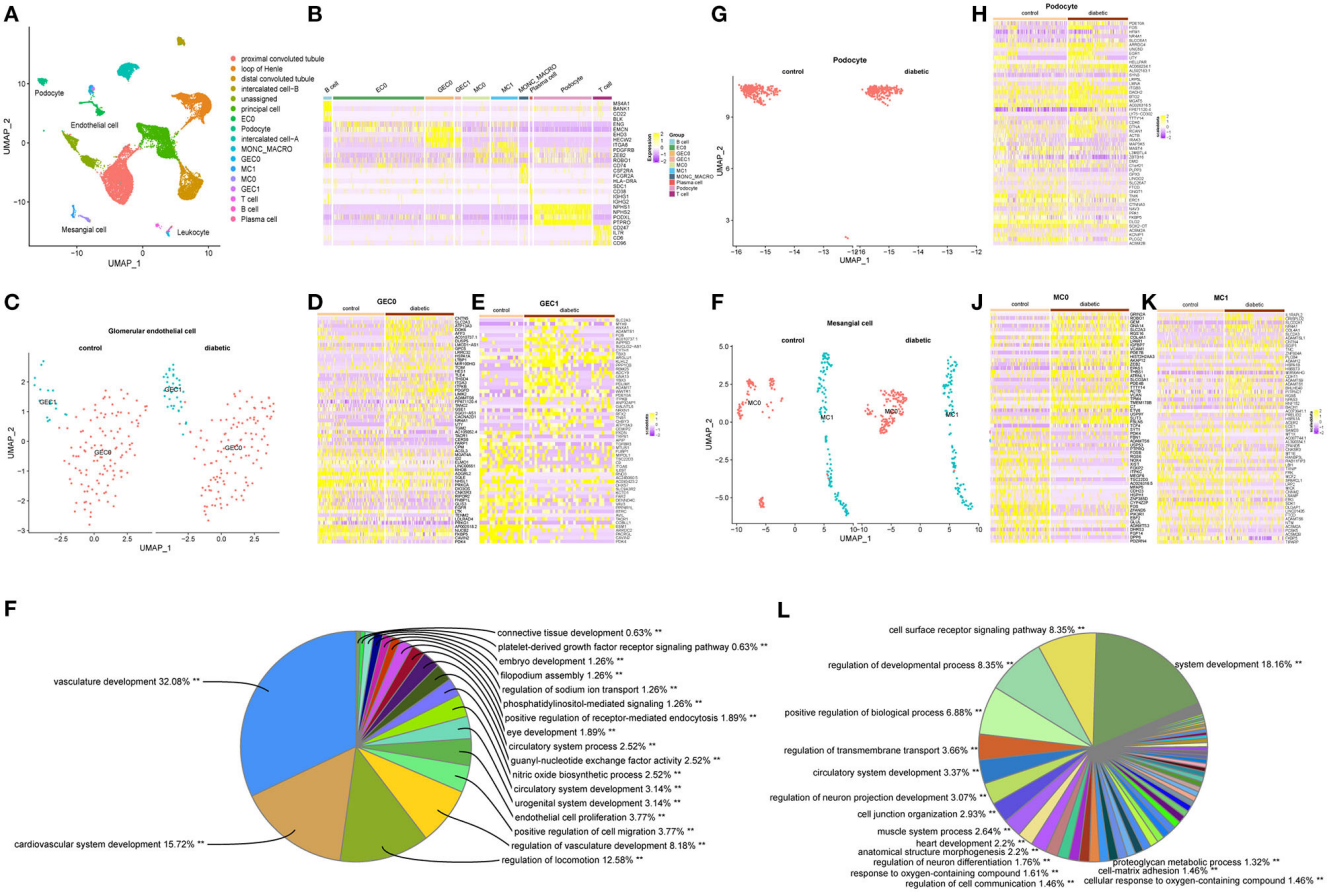
**(A)** UMAP plot from unsupervised clustering of 23,980 cells from kidney cortex of healthy subjects and patients with diabetic nephropathy. **(B)** Expression levels of representative genes from each cluster. **(C)** UMAP plot of the two subclusters of glomerular endothelial cells. **(D)** Heatmap of top 30 up- and down-regulated DEGs in GEC0. **(E)** Heatmap of top 30 up- and down-regulated DEGs of GEC1. **(F)** Pie chart of significantly enriched GO terms group of GEC1. **(G)** UMAP plot of podocytes in healthy and diabetic conditions. **(H)** Heatmap of DEGs of podocytes. **(I)** UMAP plot of the two subclusters of mesangial cells. **(J)** Heatmap of top 30 up- and down-regulated DEGs in MC0. **(K)** Heatmap of top 30 up- and down-regulated DEGs in MC1. **(L)** Pie chart of significantly enriched GO terms group from DEGs of MC1. *P*-value < 0.05 were considered statistically different.

The dataset (GSE131882) (28) were downloaded from the Gene Expression Omnibus (https://www.ncbi.nlm.nih.gov/geo/). As reported, the GSE131882 recruited three healthy and three patients with early diabetic nephropathy, among which two of the three patients presented proteinuria and glomerulosclerosis (28). The raw data were processed with zUMI (29). After gene name conversion, the Seurat package (version 3.1.2) with min.cells = 3 and min.features = 200 was used (30, 31). Quality control was performed in counts nuclei gene between 500 and 3,000, and mitochondrial gene percentage <20%. Uniform manifold approximation and projection (UMAP) presented 17 clusters of cells with dims = 1:30 and reduction = “pca.” Run Principal Component Analysis (RunPCA) was set with npcs = 50. *t*-Distributed stochastic neighbor embedding (TSNE) was generated by RUNTSNE being set with dims = 1:30 and reduction = “pca.” Nearest-neighbor search was run with FindNeighbors set with dims = 1:30. Clusters of cells were identified with the FindClusters being run with resolution = 0.1. Differential expressed genes (DEGs) among individual clusters were detected with FindAllMarkers function with the following settings: log-fold change.threshold = 0.25, min.pct = 0.1 and test.use = “wilcox.” Highly expressed genes were identified by adjusted *p*-value < 0.05 with FDR <0.05. Cluster assignment was performed based on expressions of canonical marker genes. Cell identification was performed based on previous reports (32–34) and the CellMarker database (35). A total of 19,700 cells were identified in the renal cortex, including proximal convoluted tubule cells, cells in the loop of Henle, distal convoluted tubule cells, intercalated cells, principal cells, endothelial cells, podocytes, mesangial cells, and leukocytes (Figure 1A).

Genomic data of endothelial cells, podocytes, mesangial cells, and immune cells were extracted by the Subset function in Seurat since the present study focused on cellular crosstalk inside glomeruli. RunPCA function set with npcs = 50 was used to identify significant principal components (PCs). Significant PCs was then inputted for running the RUNTSNE and RUNUMAP. For endothelial cells, FindNeighbors was run with dims = 1:10 and FindClusters was run with resolution = 0.1. For glomerular endothelial cells (36, 37), FindNeighbors was run with dims = 1:10 and FindClusters was run with resolution = 0.3. For mesangial cells, FindNeighbors was run with dims = 1:10 and FindClusters was run with resolution = 0.05. For leukocytes, FindNeighbors was run with dims = 1:20 and FindClusters was run with resolution = 0.35. Highly expressed genes were identified by adjust *p*-value < 0.05 with FDR <0.05. Cluster assignment was performed based on expression of canonical marker genes (Figure 1B).

By using the FindMarkers function with log-fold change.theshold = 0.25, min.pct = 0.1 and test.use = “t,” DEGs were defined when absolute foldchange was higher than 1.5 or lower than 0.67 with a *p*-value < 0.05.

ClueGO (38), a plug-in in Cytoscape 3.8.3, was used for DEGs enrichment. Function clusters were calculated using kappa-score on their biological roles and presented in pie charts. The top sixty DEGs, including upregulated and downregulated ones, were visualized by heatmaps and processed using the *ComplexHeatmap* R package (version 2.2.0) (39).

To study cell-to-cell communications inside glomeruli, a ligand-receptor interaction analysis was performed using iTALK (40). DEGs described above were inputted to the FindLR function and presented by the LRPlot function.

## Results

Using the pan-endothelial markers EMCN and ENG, 1,070 cells were identified as endothelial cells (41). Among them, 294 endothelial cells had high expressions of EHD3 and HECW2 and they were defined as glomerular endothelial cells (36, 37). In addition, a total of 498 podocytes, 465 mesangial cells, and 336 immune cells were detected (Table 1 and Figures 1A,B).

**Table 1:** Cell counts in glomeruli from healthy subjects and patients with diabetic nephropathy.

		**Healthy**	**Diabetic**	**Total**
Endothelial cells	GEC0	120	122	242
	GEC1	17	35	52
Podocytes	Pod	274	224	498
Mesangial cells	MC0	101	134	235
	MC1	121	109	230
Immune cells	Monocyte	19	60	79
	T cell	16	145	161
	B cell	1	71	72
	Plasma cell	0	24	24

Among the 294 glomerular endothelial cells, 137 cells were from healthy subjects, and 157 cells were from diabetic patients. Glomerular endothelial cells were further classified into GEC0 and GEC1 subsets according to the function analysis: the former having the DEGs for the negative regulation of cell activation, cell adhesion and lymphocyte activation, and positive regulation of smooth muscle proliferation (Supplementary Figure 1A); and in the latter subset, the DEGs were in the modules of vasculature development, cell migration, and endothelial cell proliferation modules in the functional analysis (Figures 1C–F and Supplementary Figure 1B). In the GEC0 cluster, 120 cells were from control subjects, and 122 cells were from diabetic patients (122 cells). In GEC1 clusters, 17 endothelial cells were from the control subjects, and 35 were from diabetic patients.

All podocytes were in one group, 274 from control and 224 from diabetes. Fifty-two DEGs were identified by comparing podocytes from the control and diabetic groups. Functional analysis revealed that the DEGs were enriched in modules of structure constituent of postsynapse, striated muscle cell apoptotic process, and skeletal muscle cell differentiation (Figures 1G,H and Supplementary Figure 1C).

Mesangial cells were grouped into MC0 and MC1 subsets. Mesangial cells in MC0 (101 in control and 134 in diabetes) were enriched for regulating anatomical structure morphogenesis, cell migration, and extracellular matrix organization (Supplementary Figure 1D), whereas MC1 cells (121 in control and 109 in diabetes) were enriched for the regulation of collagen biosynthetic process and vascular development (Figures 1I–L and Supplementary Figure 1E).

**Figure 2:**
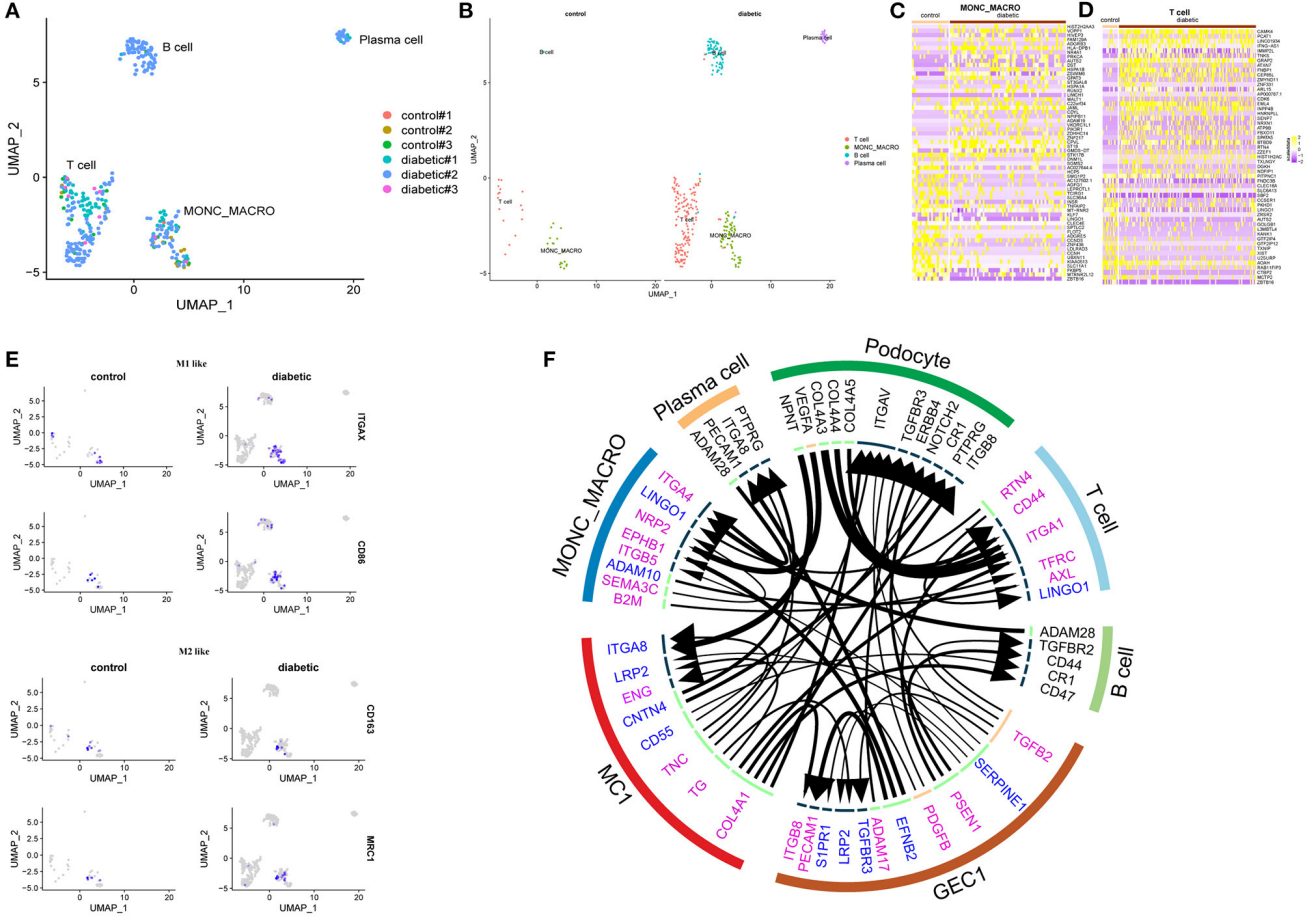
**(A)** UMAP plot of the distribution of immune cells in different samples. **(B)** UMAP plot of four subclusters of immune cells. **(C)** Heatmap of top 30 up- and down-regulated DEGs in monocytes/macrophages. **(D)** Heatmap of top 30 up- and down-regulated DEGs in T lymphocytes. **(E)** Distribution of selected cell markers of monocyte/macrophage: (Upper) Presence of M1-like macrophage markers (ITGAX and CD86) and (Lower) M2-like macrophage markers (CD163 and MRC1). **(F)** Ligand-receptor interactions prediction network with DEGs from GEC1, MC1, monocytes/macrophages (MONO_MACRO), T lymphocytes, and top 500 expressed genes of podocytes, B lymphocytes, and plasma cells. In the circus, upregulated genes are labeled purple; downregulated genes labeled blue; top expressed genes in podocytes, B lymphocytes and plasma cells labeled black. The lines and arrowheads inside are scaled to indicate the correlations of the ligand and receptor. *P*-value < 0.05 were considered statistically different.

In the cluster of immune cells, 36 cells were from control subjects, and 300 cells were from diabetic patients. Subcluster analysis further grouped the immune cells into monocytes (macrophages) which showed high expression of CD74, CSF2RA, FCGR2A, and HLA-DRA; T lymphocytes, being highly expressed with CD247, IL7R, CD6, and CD96; B lymphocytes, with high expression of MS4A1, BANK1, CD22, and BLK; as well as plasma cells which were highly expressed with SDC1, CD38, IGHG1, and IGHG2. In the monocyte (macrophage) cluster, M1-like genes, including ITGAX and CD86, and M2-like genes, including CD163 and MRC1, were presented in both control and diabetic groups (Figures 2A–E).

There were 17 T lymphocytes from the control and 145 cells from the diabetic groups. DEGs in T lymphocytes were involved in gene expression, mRNA metabolic process, and T cell receptor signaling pathway.

Seventy-one B lymphocytes (only one of them were from the control group) and 24 plasma cells (all from diabetic patients) were observed in diabetic patients with proteinuria. Top genes in B lymphocytes encoded for proteins for regulating B cell differentiation and proliferation, and B receptor signaling pathway. Top genes in plasma cells were enriched in the modules of immunoglobulin biosynthetic process and B cell receptor signaling pathway (Supplementary Figures 1F–I).

### Cell-to-Cell Communication

To study ligand-receptor interactions, DEGs of GEC1, MC1, monocytes (macrophages), T lymphocytes, as well as the top 500 genes from podocytes, B lymphocytes, and plasma cells were used. A total of 43 interactions, in autocrine and/or paracrine mechanism, were identified. In glomerular endothelial cells, TGFB2 were upregulated in diabetic group; it acted on TGFBR3 in podocytes and endothelial cells, TGFBR2 in B lymphocytes and LRP2 in mesangial cells. The growth factor PDGFB was also differentially upregulated in glomerular endothelial cells; it acted on ITGAV in podocytes and sphingosine 1-phosphate receptor 1 (S1PR1, also known as EDG1) in endothelial cells. The upregulated molecule ADAM17 in glomerular endothelial cells affected ERBB4 in podocytes, and the upregulated PSEN1 acted at CD44 of B lymphocytes and T lymphocytes and NOTCH2 of podocytes. Increased expression of ITGB8 were also observed in glomerular endothelial cells, and this protein responded to COL4A1 from mesangial cells. The receptor for TGFB2 from endothelial cells, LRP2, were downregulated in both endothelial and mesangial cells of the diabetic patients. ADAM28 was downregulated in plasma cells and B cells (Figure 2F).

## Discussion

By performing bioinformatics analysis on the online single-nucleus RNA sequencing dataset regarding glomerular cells in diabetic patients (28), the present study reports that (1) glomerular endothelial cells also play a critical role in the development of diabetic nephropathy; (2) apart from well-studied diabetes/high glucose-induced endothelial dysfunction, a group of glomerular endothelial cells possesses characteristic angiogenesis genes; and (3) immune cells such as macrophages, T lymphocytes, B lymphocytes, and plasma cells take part in the progress of diabetic nephropathy.

Podocytes and podocyte-released glomerular basement membrane are critical for preventing macro-molecular proteins from filtering out from the plasma to the kidney tubules. Moreover, podocytes are an important cell source of growth factors, which regulate endothelial cell proliferation and angiogenesis. In the present study, VEGFA, EGR1, and NOTCH2 genes are among the top 500 highly-expressed genes, supporting the critical role of podocytes in maintaining glomerular endothelial hemostasis. It is reported that podocyte counts increase in the early stage and decrease in the advanced stage of diabetes (42, 43). The present study identified comparable podocyte counts between the control and diabetic groups, with the latter showing fifty-two DEGs, in which none of them were enriched in modules of angiogenesis, vascular development, or glomerular development. The findings thus suggest that the molecular and functional changes in podocytes unlikely contribute to the progress, at least, not during the initiation phase of diabetic nephropathy.

ADAM metallopeptidase domain 17 (ADAM17) is a disintegrin and metalloprotease. By shedding tumor nuclear factor, platelet receptors glycoprotein 1, adhesion molecules, and angiotensin-converting enzyme converting enzyme 2 (ACE2), ADAM17 plays a critical role in the proinflammatory responses, thrombus formation, and renin-angiotensin system activation (44–47). ADAM17 and its shedding effects on ACE2 lead to glomerular area enlargement, glomerular and tubular basement membrane thickening, mesangial matrix expansion, and collagen deposition (48). Increased expressions of ADAM17 in kidneys are reported in diabetic patients (49) and experimental diabetic rodents (50–52). In the present study, ADAM17 in glomerular endothelial cells was upregulated, and it targeted at V-Erb-B2 avian erythroblastic leukemia viral oncogene homolog 4 (ERBB4), a member of the epidermal growth factor receptor family (EGFR), in the podocytes. Increased ERBB4 expressions phosphorylate EGFR, activates TGF-Smad-2/3 signaling, resulting in podocyte apoptosis in type 2 diabetic patients and diabetic mice (both type 1 and type 2) (53). Blockade of ERBB4 reduces glomerular damage and protects animals from the development of albuminuria (54, 55).

The role of glomerular endothelial dysfunction in the initiation and development of diabetic nephropathy has drawn attention recently. Besides well-studied endothelial dysfunction in macrocirculation in diabetes (56–59), characterized by reduced nitric oxide bioavailability, increased oxidative stress, and enhanced inflammatory responses, endothelial cells in the microcirculation also present upregulation of adhesion molecules, breakdown of endothelial barrier, and aberrant angiogenesis. Angiogenesis is a characteristic feature in diabetic microcirculation (57). The present analysis demonstrates that diabetic patients have a higher proportion of glomerular endothelial cells in the GEC1 group, the glomerular endothelial cells with high expression of angiogenetic genes, than control subjects (17/137 for healthy subjects, and 35/157 for diabetic patients), thus supporting that diabetes induces glomerular endothelial cell proliferation, and these proliferative endothelial cells are fundamental for immature angiogenesis, vessel leakage as well as glomerulosclerosis.

VEGF, an endothelial-specific growth factor, promotes endothelial cell proliferation and differentiation, resulting in increased endothelial permeability. Under physiological conditions, a low basal VEGF level is required for endothelial cell homeostasis (60). VEGF, mainly VEGFA, is produced by podocytes, and VEGFRs are present on glomerular endothelial cells. In the present study, VEGFA was highly expressed in podocytes while an upregulation of its canonical receptors in glomerular endothelial cells was not detected in diabetic patients; the finding thus suggests that other angiogenetic signaling pathways are involved in diabetes-induced aberrant angiogenesis.

Endothelial released-PDGFB, another angiogenetic factor, targeted S1PR1 in endothelial cells, which was downregulated in the present study. The S1PR1, a G-protein-coupled receptor family member, responds to sphingosine-1-phosphate (S1P) (61), VEGF (62), and PDGFB (63). S1PR1 is mainly expressed in microvascular endothelial cells and plays a critical role in promoting barrier integrity (64, 65), sproutings (62), angiogenesis maturation (66–68), and nitric oxide generation (69). Endothelium-specific S1PR1-knockout mice exhibit impaired blood-brain-barrier integrity and increased adhesion molecule expressions in a middle cerebral artery occlusion-induced stroke model (70–72). Cardiomyocyte-restricted deletion of S1PR1 shows progressive cardiomyopathy and premature death due to impaired activity of sarcolemmal Na^+^/H^+^ exchange and increased Ca^2+^ sensitivity (73). S1PR1 signaling pathway controls the renal vasculature development in mouse early embryogenesis (74), and protects glycocalyx by shedding syndecan-1 (75). The uncoupled expressions of PDGFB and S1PR1, with the former being upregulated and the latter downregulated, in the present study and literatures (63) indicates that the overspilled PDFGB probably signals through other receptors, resulting in endothelial barrier leakage and immature angiogenesis.

Ephrin B2 (EFNB2) was decreased in endothelial cells, while its receptor EPHB1 was increased in monocytes/macrophages. Ephrin/Eph receptor interactions are bidirectional and play essential roles in vascular development. Mice with endothelial EFNB2-deletion display a severely compromised vascular system and die at mid-gestation. Inhibiting Ephrin B ligands prevents endothelial cell sprouting and induces endothelial cell assembly in disorder (76–79). Besides, Ephrin/Eph receptor interaction facilitates macrophage recognition of differentiating human erythroblasts (80).

Serpin family E member 1 (SERPINE1, also known as endothelial plasminogen activator inhibitor PAI-1) is the primary physiological inhibitor of tissue plasminogen activator and urokinase-type plasminogen (uPA) activator, and participates in preventing fibrinolysis and promoting angiogenesis as well as inhibiting matrix metalloproteinases (81, 82). SERPINE1 stimulates angiogenesis through its vitronectin-binding function. SERPINE1 promotes angiogenesis at physiological concentrations but inhibits vascularization at pharmacological concentrations (83). By combining with uPA receptor and LDL-receptor-associated protein (LRP), SERPINE1 affects monocyte/macrophage motility (84–86). In the present study, both glomerular endothelial cells and mesangial cells have reduced expressions of LRP2, an endocytic receptor for protein reabsorption from the glomerular filtrate. So far, the presence of LRP is mainly reported in tubular cells and podocytes, with few reports in mesangial cells and glomerular endothelial cells.

Presenilin-1 (PSEN1) is a component of synaptic and endothelial adherens junctions (87). Genetic mutation on presenilin-1 presents early-onset Alzheimer symptoms in mice, accompanied by decreases in capillary sprouting sites and increases in capillary diameter (88). It indicates that PSEN1 is involved in angiogenesis.

In the high-angiogenetic GEC1 group, increased expressions of PDGFB, TGFB2, ADAM17, and ITGB8, and reduced expression of S1PR1 are linked to glomerular angiogenesis and glomerulosclerosis, whereas the increased expression of presenilin-1 and the decreased expressions of SERPINE1 and EFNB2 in glomerular endothelial cells correlate to the downregulation of angiogenesis. The activity of glomerular endothelial cells, including immature angiogenesis, is regulated by podocytes, mesangial cells, glomerular endothelial cells, and immune cells in a paracrine and/or an autocrine way. The co-existence of pro-angiogenetic and anti-angiogenetic factors in glomeruli of diabetic patients indicates that diabetes-induced angiogenesis is counterbalanced by the compensatory mechanisms from the neighboring cells. It is further confirmed with their pathological changes that two of three diabetic patients presented with proteinuria and an increased proportion of global glomerulosclerosis (28). It is important to note that the progressive changes in diabetic nephropathy are hard to restore when compensatory works fade. Therefore, protecting endothelial cell function and preventing angiogenesis may have therapeutic potential since the two compensatory molecules have additional physiological roles.

In addition to aberrant angiogenesis, interstitial fibrosis is another characteristic feature of diabetic nephropathy. Integrins are a family of ubiquitous αβ heterodimeric receptors. Integrins form receptors for different ligands due to combinations of alpha and beta subunits; thus, one integrin binds several ligands while one ligand is recognized by several integrins (89–91). Integrins regulate a variety of biological processes, including cell growth, proliferation, migration, signaling, and cytokine activation, thereby playing important roles in inflammation, infection, and angiogenesis (92). In glomeruli, integrin α8 (ITGA8) is exclusively present in mesangial cells (93, 94). Increased ITGA8 expression has the potential to be a clinical marker of glomerular disease prognosis since ITGA8 supports adhesion of mesangial cells (95), reduces cell proliferation (96), protects against apoptosis (97), and facilitates phagocytosis (95, 98). Of importance, increased expressions of ITGB8 play a role in glomerular endothelial viability by controlling the release of bioactive TGF-β (99, 100), a potent inducer of endothelial-mesenchymal transition, especially TGF-β2 isoform (101). In the present study, ITGB8/ITGA8 was on the top list of the genes expressed in podocytes and plasma cells. While ITGB8 expression is increased in glomerular endothelial cells, ITGA8 expression is decreased in mesangial cells. Moreover, COL4A1 in mesangial cells, the expression of which was increased in diabetes, acted on ITGB8 in glomerular endothelial cells. The upregulated TGF-β2 in endothelial cells further activates its corresponding receptors, namely TGFBR2, TGFBR3 and ENG, on B lymphocytes, podocytes, glomerular endothelial cells, and mesangial cells, respectively, leading to epithelial-mesenchymal transition and fibrosis in the development of diabetic nephropathy.

In addition, ITGA1 expression was increased in T lymphocytes, and ITGA4 expression was on the top gene list of monocytes/macrophages. Blocking ITGA4 inhibits neutrophil migration into the glomerulus and reduces proteinuria in mice with glomerular basement membrane nephritis (102). Combined treatment of anti-ITGB2 and anti-ITGA4 antibodies reduces monocyte/macrophage infiltration into the glomeruli, while neither alone has significant effects (103).

Both integrin and CD44 respond to osteopontin, collagens, and matrix metalloproteinases (104). In the present study, CD44 expressions were upregulated in both T and B lymphocytes, suggesting that glomerular parenchymal cells, together with lymphocytes, participate in glomerulosclerosis.

Semaphorins are a large family of secreted and membrane-bound proteins. The class 3 secreted semaphorin, SEMA3, is present in human peripheral blood monocytes. In response to signals of cadherins (105, 106), and VEGFRs (107, 108), SEMA3 forms complexes with neuropilin (NRP) and integrins (109, 110) to regulate organ development, tissue repair, immune responses, and tumorigenesis processes (109, 111–113). Both NRP1 and NRP2 expressions are reduced in M1 differentiation, while NRP1 and SEMA3A expression are increased in M2 phenotype (114). Concomitant upregulation of SEMA3A and NRP2 demonstrated in the present analysis indicates that diabetes induces M2-like macrophages through an autocrine mechanism (115).

ADAM28 expression is downregulated in both B lymphocytes and plasma cells. ADAM28 expression is positively related to B cell proliferation (116). Upregulated CD19 controls B cell differentiation by regulating ADAM28-mediated NOTCH2 cleavage (117). It indicates that these antibody-producing lymphocytes are inactivated or dysfunctional in diabetes, although presences of B lymphocytes and plasma cells were exclusively observed in diabetes.

By analyzing the online dataset GSE131882 (28), the present study focuses on exploring the interactions of parenchymal cells of glomeruli as well as the potential involvements of immune cells in the progress of diabetic nephropathy, and special attention is paid to the angiogenesis process. Both pro- and anti-angiogenetic genes are observed in the GEC1 and its neighboring cells, indicating a dynamic interplay between parenchymal cells and immune cells in the glomerulus during the early stage of the disease. A limitation of this study is a lack of confirmation in a cohort of diabetic kidneys. Given the reluctance of diabetic patients for biopsy, pertinent experimental animal models are an alternative. However, species differences are a critical issue, because species-specific genes and cell-type identification can affect the analysis. In human diabetes, endothelial cells with high expression of EHD3 and HECW2 are defined as glomerular endothelial cells; by contrast, in mouse diabetes, endothelial cells are identified with high expression of Emcn, Kdr, Flt1, and Pecam1 (36). Thus, cautions are warranted for the interpretation, as well as conclusion, of dataset from experimental animals, and a direct extrapolation of those dataset to human condition may not be feasible.

In brief, the present study reports comprehensive interactions in diabetic glomeruli. A subgroup of glomerular endothelial cells with pro-angiogenesis characteristics is identified, thereby providing an evidence for the critical contribution of immature angiogenesis to the vessel leakage, glomerular barrier dysfunction, and glomerulosclerosis in the progress of diabetic nephropathy. Furthermore, glomerular endothelial cells are not an independent player in the progress of diabetic nephropathy. Inside glomeruli, podocytes, mesangial cells, monocytes/macrophages, lymphocytes are all orchestrating in the scenario. The identification of glomerular endothelial cells with angiogenetic characters and the signaling pathways involved in the present study shed light on the therapeutic target for diabetic nephropathy.

## Data Availability Statement

The datasets presented in this study can be found in online repositories. The names of the repository/repositories and accession number(s) can be found in the article/Supplementary Material.

## Author Contributions

TL, KS, and JL analyzed the data. TZ, SL, and YS wrote the manuscript, read, edited/revised the manuscripts, and gave final content approval. All authors contributed to the article and approved the submitted version.

## Conflict of Interest

The authors declare that the research was conducted in the absence of any commercial or financial relationships that could be construed as a potential conflict of interest.

## Supplementary Material

Supplementary Figure 1 **(A)** Pie chart of functional biological modules (left) and GO terms (right) of DEGs in GEC0. **(B)** Pie chart of functional biological modules (left) and GO terms (right) of DEGs in GEC1. **(C)** Pie chart of functional biological modules (left) and GO terms (right) of DEGs in podocytes. **(D)** Pie chart of functional biological modules (left) and GO terms (right) of DEGs in MC0. **(E)** Pie chart of functional biological modules (left) and GO terms (right) of DEGs in MC1. **(F)** Pie chart of functional biological modules (left) and GO terms (right) of DEGs in monocytes/macrophages. **(G)** Pie chart of functional biological modules (left) and GO terms (right) of DEGs in T lymphocytes. **(H)** Pie chart of functional biological modules (left) and GO terms (right) of top 200 genes in B lymphocytes. **(I)** Pie chart of functional biological modules (left) and GO terms (right) of top 200 genes in plasma cells.
