# Supplementary figures and images for: Glomerular Endothelial Cells Are the Coordinator in the Development of Diabetic Nephropathy
Source: Front Med (Lausanne). 2021 Jun 18;8:655639. doi: 10.3389/fmed.2021.655639 (PMC8249723; doi:10.3389/fmed.2021.655639)

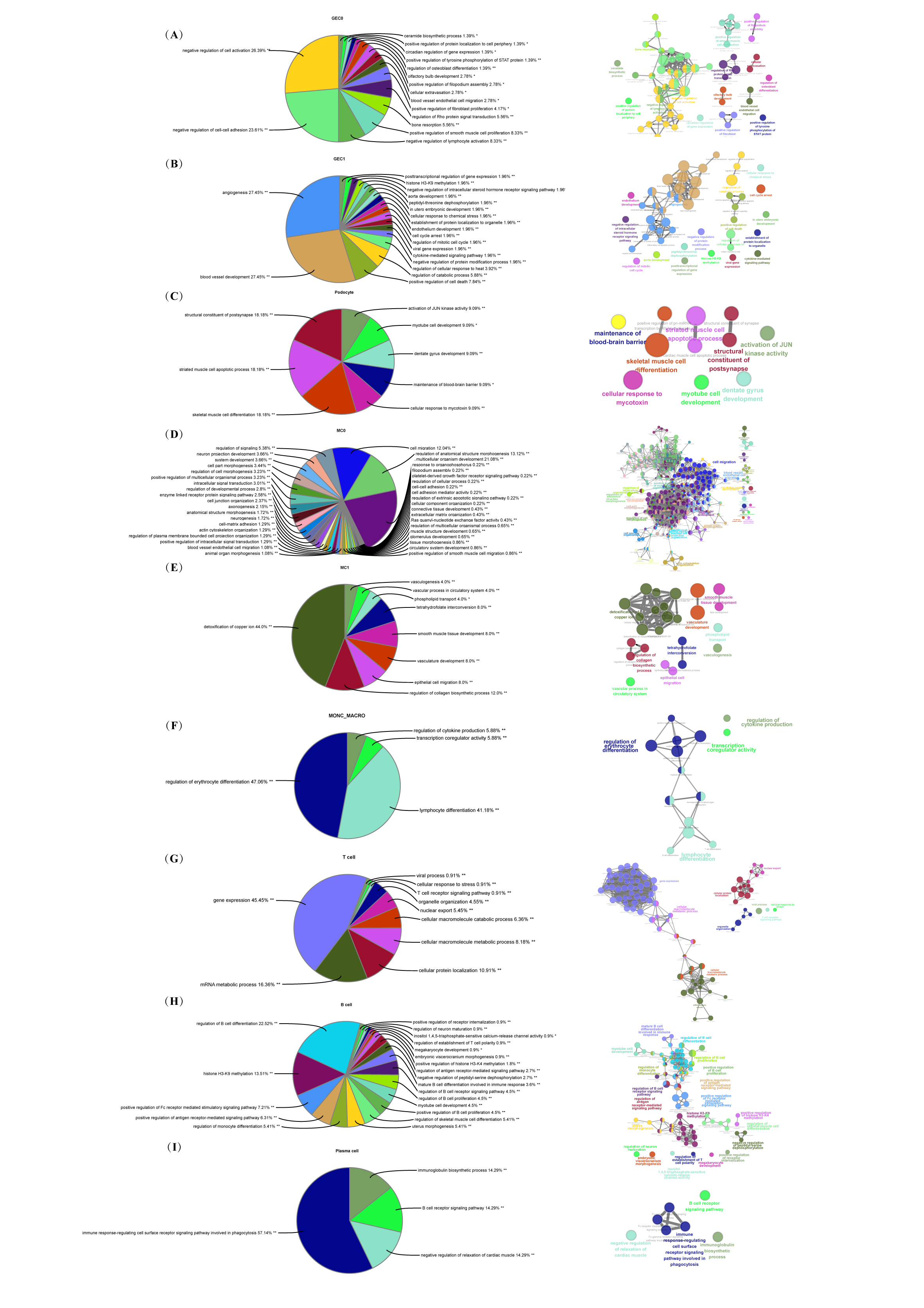

Supplement: Supplementary Figure 1 — (A) Pie chart of functional biological modules (left) and GO terms (right) of DEGs in GEC0. (B) Pie chart of functional biological modules (left) and GO terms (right) of DEGs in GEC1. (C) Pie chart of functional biological modules (left) and GO terms (right) of DEGs in podocytes. (D) Pie chart of functional biological modules (left) and GO terms (right) of DEGs in MC0. (E) Pie chart of functional biological modules (left) and GO terms (right) of DEGs in MC1. (F) Pie chart of functional biological modules (left) and GO terms (right) of DEGs in monocytes/macrophages. (G) Pie chart of functional biological modules (left) and GO terms (right) of DEGs in T lymphocytes. (H) Pie chart of functional biological modules (left) and GO terms (right) of top 200 genes in B lymphocytes. (I) Pie chart of functional biological modules (left) and GO terms (right) of top 200 genes in plasma cells. [file Image_1.TIF]
